# Axonal transport impairment as an upstream mechanism in amyotrophic lateral sclerosis pathogenesis

**DOI:** 10.3389/fnins.2026.1802313

**Published:** 2026-03-11

**Authors:** Uri Gabbay

**Affiliations:** 1 Gray Faculty of Medicine, Tel Aviv University, Tel Aviv-Yafo, Israel; 2 Quality Unit, Rabin Medical Center, Petah Tikva, Israel

**Keywords:** amyotrophic lateral sclerosis, axonal transport, biomarkers, neurodegeneration, neuromuscular junction

## Abstract

Amyotrophic lateral sclerosis (ALS) is a fatal neurodegenerative disorder characterized by progressive loss of upper and lower motor neurons. Despite marked genetic and pathological heterogeneity, a unifying pathogenic framework remains lacking. We propose that axonal transport impairment represents an early and convergent but genotype-modulated upstream vulnerability in ALS, contributing to distal synaptic failure, bioenergetic stress, protein aggregation, neuroinflammation, and neuronal death. Across many ALS models, including SOD1, TARDBP (TDP-43), FUS, and C9orf72, transport deficits are frequently detectable in presymptomatic stages, often preceding overt motor neuron loss or clinical manifestation, although temporal ordering varies by molecular subtype. Human data from induced pluripotent stem cell-derived motor neurons and neuroimaging in mutation carriers further support early transport dysfunction in both familial and sporadic ALS. We synthesize genetic, cellular, and systems-level evidence demonstrating that diverse ALS-associated mutations converge on intracellular trafficking machinery through distinct but interacting mechanisms, disrupting long-range cargo delivery and clearance in motor neurons. This framework provides a mechanistic basis for selective motor neuron vulnerability, the dying-back pattern of neuromuscular junction degeneration, and the emergence of downstream pathological hallmarks including mitochondrial dysfunction, excitotoxicity, aggregation, and inflammation. This model generates testable predictions regarding presymptomatic transport biomarkers and the timing of therapeutic intervention. We discuss implications for biomarker development and therapeutic strategy, proposing restoration of axonal transport as a central component of rational multimodal disease modification in ALS.

## Introduction

Amyotrophic lateral sclerosis (ALS) is a relentlessly progressive neurodegenerative disorder that leads to paralysis and death within 3–5 years of symptom onset. Although more than 40 genes have been implicated in ALS, including *SOD1, TARDBP, FUS*, and *C9orf72*, no single pathogenic pathway has unified this genetic diversity into a coherent disease model (Goutman et al., 2022a; Feldman et al., 2022; Mizielinska et al., 2025). Pathologically, ALS is characterized by distal axon degeneration, neuromuscular junction (NMJ) denervation, protein aggregation, mitochondrial dysfunction, and neuroinflammation (Goutman et al., 2022a; Feldman et al., 2022; Verma et al., 2022). Whether these processes represent primary drivers or downstream consequences of disease remains unresolved.

A defining biological feature of motor neurons is their extreme morphological polarization. Lower motor neurons extend axons up to 1 meter from the spinal cord to muscle, while corticospinal motor neurons project from the motor cortex to the spinal cord, imposing substantial logistical demands on intracellular trafficking (Feldman et al., 2022; Nijssen et al., 2017). These neurons rely critically on microtubule-based transport systems to deliver mitochondria, mRNAs, synaptic vesicles, and trophic signaling endosomes to distal compartments and to return damaged cargo for degradation (Burk and Pasterkamp, 2019; Berth and Lloyd, 2023; De Vos and Hafezparast, 2017). Disruption of this transport network is therefore expected to predispose distal axons to bioenergetic stress, synaptic instability, and progressive dying-back degeneration.

Most ALS animal models are mutation-driven (e.g., *SOD1, FUS, TARDBP, C9orf72*), whereas most human ALS are sporadic and multifactorial. Disease onset and progression in animals are typically faster and genetically constrained; therefore, mechanistic inferences must be translated cautiously to human ALS (Goutman et al., 2022a).

Despite extensive mechanistic heterogeneity, multiple ALS-associated pathways converge on biological processes critically dependent on efficient long-range intracellular trafficking. However, no unifying framework has systematically examined whether axonal transport impairment constitutes a temporally upstream vulnerability linking these diverse molecular mechanisms. This perspective therefore considers the possibility that disrupted axonal transport represents a shared pathogenic bottleneck connecting genetic risk to selective motor neuron degeneration.

Here we argue that axonal transport impairment represents an early and convergent, but genotype-modulated upstream vulnerability in ALS. Rather than being solely secondary to protein aggregation or mitochondrial dysfunction, transport failure emerges in many experimental systems prior to overt neuronal loss and provides a mechanistic bridge linking genetic risk to distal synaptic failure and motor neuron degeneration. As a Perspective article, this manuscript presents a focused, hypothesis-driven synthesis rather than a comprehensive systematic review.

## Evidence supporting the temporal primacy of axonal transport impairment

Axonal transport defects have been shown to precede neurodegeneration in many ALS genotypes, including TDP-43-associated disease, although this temporal ordering is not universal across all molecular subtypes (Sleigh et al., 2020). Presymptomatic axonal transport deficits are among the earliest pathological features detected in several ALS models. In SOD1(G93A) transgenic mice, impaired retrograde axonal transport is detectable *in vivo* before the onset of weakness, motor neuron loss, or neuromuscular junction (NMJ) denervation (Bilsland et al., 2010). These deficits worsen during early symptomatic stages, indicating progressive deterioration of the transport machinery. Consistent with this, microfluidic analysis of cultured motor neurons from SOD1 mice reveals abnormal transport of mitochondria and acidic vesicles prior to axonal degeneration (Otomo et al., 2022).

TDP-43-linked ALS shows a similar temporal pattern. Mice carrying the TDP-43(M337V) mutation display defects in axonal transport of signaling endosomes between 1.5 and 3 months of age, well before symptom onset and in the absence of motor neuron loss (Sleigh et al., 2020). In human ALS, hyperphosphorylated TDP-43 accumulates within axons and intramuscular nerve bundles of presymptomatic patients, where it disrupts local translation and microtubule-dependent trafficking through formation of aberrant ribonucleoprotein granules (Balendra et al., 2025; Ionescu et al., 2023; Kurashige et al., 2022). These findings support early disturbance of axonal trafficking processes in both experimental and human disease.

Importantly, the temporal relationship between transport dysfunction and neurodegeneration varies across genetic subtypes. Sleigh et al. (2020) demonstrated that retrograde endosomal transport is impaired before symptom onset in TDP-43(M337V) mice but remains preserved in FusΔ14/+ mice despite comparable motor neuron loss, demonstrating genotype-specific temporal ordering.

Despite this heterogeneity, multiple experimental systems converge on axonal transport vulnerability. Drosophila models expressing mutant SOD1, TDP-43, FUS, or C9orf72 exhibit transport abnormalities affecting distinct cargoes but impairing long-range trafficking (Baldwin et al., 2016). Human motor neurons derived from sporadic ALS patient iPSCs likewise show marked axonal transport defects associated with dysregulated axon guidance pathways, indicating that transport impairment is not restricted to familial disease (Ye et al., 2025).

Collectively, these findings support the interpretation that axonal transport impairment is a presymptomatic and progressive feature in the majority of ALS experimental models studied to date and may represent a convergent, genotype-modulated upstream vulnerability across genetically diverse disease mechanisms.

## Genetic convergence on axonal transport machinery

Despite substantial genetic heterogeneity, ALS shows striking convergence on intracellular trafficking pathways. Several ALS-associated genes encode proteins that regulate key structural or functional components of the axonal transport system. Mutations in TUBA4A, PFN1, DCTN1, and KIF5A alter microtubule stability, actin dynamics, or motor protein function, impairing the efficiency of cargo movement along axons (Goutman et al., 2022a; Feldman et al., 2022).

Other ALS genes disrupt transport indirectly through dysregulation of RNA metabolism and local translation. TDP-43 regulates axonal mRNA localization and microtubule-dependent transport; mutant TDP-43 forms aberrant ribonucleoprotein granules that interfere with cargo trafficking and inhibit local protein synthesis at distal axons and neuromuscular junctions (Balendra et al., 2025; Ionescu et al., 2023; Piol et al., 2023). FUS mutations similarly disrupt mRNA localization and synaptic maintenance, promoting neuromuscular junction instability and axonal degeneration (Piol et al., 2023; Picchiarelli et al., 2019).

C9orf72 repeat expansions impair transport through multiple mechanisms, including haploinsufficiency of the normal C9orf72 protein, which participates in vesicular trafficking and autophagy; sequestration of RNA-binding proteins into RNA foci; and production of toxic dipeptide repeat proteins that interfere with multiple cellular processes, including intracellular trafficking (Goutman et al., 2022a; Feldman et al., 2022; Mizielinska et al., 2025).

A closely related trafficking system, nucleocytoplasmic transport is also disrupted in ALS. TDP-43 aggregation, mutant FUS, and C9orf72 expansions impair nuclear pore complex integrity, leading to defective nuclear import and RNA export (Chou et al., 2018; Prpar Mihevc et al., 2017). Because axonal transport depends on continuous supply of nuclear-derived mRNAs and proteins, disruption of nucleocytoplasmic trafficking may further destabilize long-range neuronal logistics.

These convergent genetic effects are particularly consequential in motor neurons, whose extreme axonal length imposes unusually high dependence on efficient intracellular transport. Accordingly, genetically diverse ALS mutations converge on disruption of intracellular organization and trafficking processes essential for motor neuron maintenance and survival.

## Pathophysiological consequences of axonal transport impairment

**Figure 1:**
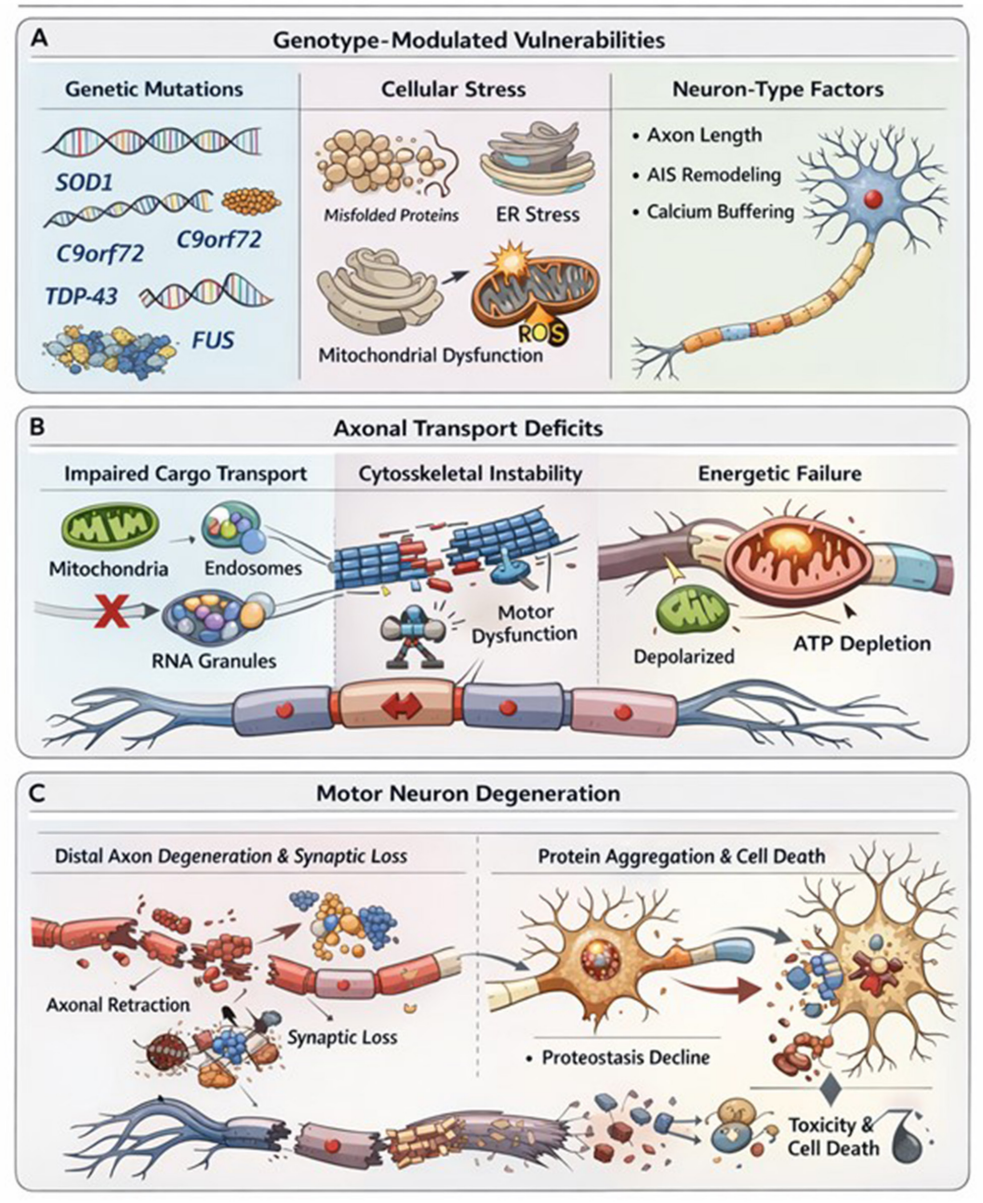
Conceptual model illustrating relationships between genetic perturbations, axonal transport disruption, and downstream cellular consequences in ALS. **(A)** ALS-associated mutations affect cytoskeletal organization, RNA metabolism, vesicular trafficking, and nucleocytoplasmic transport. **(B)** Disrupted transport reduces delivery of mitochondria, mRNAs, and trophic signals to distal axons and neuromuscular junctions. **(C)** Distal cellular stress is associated with synaptic dysfunction, mitochondrial abnormalities, protein aggregation, and inflammatory activation. Selective neuronal vulnerability reflects interactions among axon length, projection-neuron identity, metabolic demand, and intrinsic stress-response programs.

The proposed framework is summarized schematically (Figure 1), illustrating how genetically heterogeneous molecular insults may converge on axonal transport dysfunction as an early systems-level vulnerability linking distal synaptic failure to downstream neurodegenerative cascades. Axonal transport failure predicts early degeneration at the neuromuscular junction (NMJ), consistent with the well-established dying-back pattern observed in ALS (Verma et al., 2022; Coleman, 2022). Distal synapses operate near energetic and proteostatic limits and possess limited repair capacity, rendering them particularly vulnerable when intracellular supply lines are compromised.

Across ALS models, NMJ dysfunction precedes motor neuron loss. In TDP-43 transgenic mice, impaired synaptic transmission—including reduced vesicle fusion and quantal release—appears months before motor deficits or neuronal death (Chand et al., 2018). FUS-ALS knock-in mice exhibit postsynaptic NMJ defects in neonates and progressive endplate denervation in adults prior to motor neuron loss, consistent with early synaptic destabilization (Picchiarelli et al., 2019). In humans, accumulation of TDP-43 within intramuscular nerve bundles of presymptomatic patients supports early distal pathology disrupting local axonal translation and synaptic maintenance (Ionescu et al., 2023; Kurashige et al., 2022).

Selective vulnerability of fast-fatigable motor units further supports a transport-dependent model. Fast motor units show earlier and more pronounced transport deficits than slow units (Nijssen et al., 2017; Tremblay et al., 2017; Morisaki et al., 2016). In SOD1(G93A) mice, fast NMJs exhibit reduced quantal content well before motor neuron death, whereas slow units are affected later (Tremblay et al., 2017). Under physiological conditions, BDNF enhances axonal transport selectively in fast motor neurons; however, this compensatory response is lost in SOD1(G93A) neurons, which become refractory to BDNF stimulation (Tosolini et al., 2022). Fast motor neurons also express lower levels of protective factors such as osteopontin and higher levels of matrix metalloproteinase-9, increasing their susceptibility to transport-dependent degeneration (Nijssen et al., 2017; Morisaki et al., 2016). As fast units are lost, surviving units adopt faster phenotypes, potentially representing maladaptive compensation (Weddell et al., 2021).

Transport disruption also impairs mitochondrial trafficking, leading to focal bioenergetic collapse at distal axons and NMJs (Otomo et al., 2022; Yipeng et al., 2025). In SOD1 neurons, mitochondrial transport is reduced while autophagosome and late endosome trafficking is paradoxically increased, suggesting incomplete compensatory clearance (Otomo et al., 2022). Mitochondrial dysfunction at NMJs may therefore reflect impaired ATP production, calcium dysregulation, oxidative stress, and defective mitochondrial biogenesis (Yipeng et al., 2025). In C9orf72 models, activation of the PGC-1α pathway rescues both mitochondrial bioenergetics and axonal transport, demonstrating bidirectional coupling between transport and metabolic integrity (Mehta et al., 2021).

Impaired trafficking may also contribute to protein aggregation. Reduced retrograde clearance of damaged proteins and disruption of RNA-binding protein dynamics promote distal accumulation of misfolded species such as TDP-43 and FUS (Burk and Pasterkamp, 2019; Balendra et al., 2025; Ionescu et al., 2023; Piol et al., 2023). Aggregation may therefore emerge, at least in part, as a downstream consequence of impaired neuronal logistics rather than an exclusively primary event.

Local ATP depletion further impairs calcium extrusion and glutamate uptake, promoting neuronal hyperexcitability and excitotoxic stress (Feldman et al., 2022; Yipeng et al., 2025). Degenerating distal axons release damage-associated molecular patterns that activate microglia and astrocytes, suggesting that neuroinflammation may act as a secondary amplifier of disease progression rather than its initiator (Goutman et al., 2022a; Feldman et al., 2022; Verma et al., 2022).

Collectively, these findings support a model in which axonal transport impairment initiates a cascade of distal bioenergetic failure, synaptic destabilization, aggregation, excitability imbalance, and inflammatory amplification, progressively undermining motor neuron survival.

## Upper motor neuron vulnerability and long-range transport

Corticospinal motor neurons project axons from the motor cortex to the spinal cord, imposing logistical demands comparable to those of lower motor neurons (Feldman et al., 2022; Nijssen et al., 2017). Neuroimaging in presymptomatic *C9orf72* mutation carriers reveals early white-matter abnormalities in corticospinal and callosal tracts, including reduced fractional anisotropy and tract atrophy, years to decades before symptom onset (Goutman et al., 2022a; Feldman et al., 2022; Mizielinska et al., 2025). Longitudinal diffusion tensor imaging further demonstrates progressive degeneration of these tracts prior to clinical disease (Goutman et al., 2022a; Mizielinska et al., 2025).

TDP-43 pathology disrupts both nucleocytoplasmic and axonal transport in cortical neurons, with hyperphosphorylated TDP-43 accumulating in axons and impairing local translation and cargo trafficking (Balendra et al., 2025; Chou et al., 2018). Preferential loss of fast-conducting corticospinal axons, detected by transcranial stimulation and corticomuscular coherence studies, supports transport-dependent vulnerability of long-range projection neurons (Feldman et al., 2022). In primary lateral sclerosis, the most severe white-matter abnormalities occur in callosal motor fibers, accompanied by cortical inexcitability rather than hyperexcitability, suggesting that cortical-specific properties interact with transport failure to shape phenotype (Feldman et al., 2022; Alhindi et al., 2023).

Nevertheless, selective vulnerability in ALS cannot be explained by axon length alone. Although long projection neurons depend critically on efficient intracellular trafficking, intrinsic cell-type-specific properties strongly modify susceptibility to transport disruption.

Motor neurons, but not sensory neurons require proteasome-dependent remodeling of the axon initial segment (AIS) to permit increased mitochondrial entry into the axon during periods of metabolic stress. Under proteostatic conditions characteristic of ALS, this adaptive remodeling fails in motor neurons, limiting mitochondrial delivery to distal compartments and precipitating focal bioenergetic collapse at neuromuscular junctions and distal axons (Tra et al., 2025). In contrast, sensory neurons lack this AIS-dependent constraint and maintain more permissive mitochondrial trafficking under stress.

Additional molecular differences further contribute to sensory neuron resilience. Sensory neurons accumulate mutant SOD1 yet lack Derlin-1, a mediator of endoplasmic reticulum stress signaling, thereby avoiding ER-stress-mediated apoptosis despite protein accumulation (Taiana et al., 2016). Together, these findings indicate that sensory neuron sparing reflects differences in axon initial segment biology, mitochondrial trafficking capacity, and stress-response pathways rather than axon length per se (Tra et al., 2025; Taiana et al., 2016; Bombaci et al., 2023; Seki et al., 2019).

Within the motor system, vulnerability also reflects projection neuron identity. Corticospinal and corticobulbar neurons are subcerebral projection neurons (SCPNs) that share transcriptional programs and demonstrate early and selective degeneration in ALS models (Ozdinler et al., 2011). Their long-range projections, high metabolic demand, and excitability profiles amplify dependence on sustained transport fidelity.

By contrast, oculomotor neurons are relatively spared in ALS despite substantial axonal projections. Their resistance is associated with intrinsic protective programs, including enhanced calcium buffering, higher mitochondrial capacity, and greater proteostatic resilience (Verma et al., 2022; Ragagnin et al., 2019).

## Biomarker implications

Neurofilament light chain (NfL) is the most validated biomarker in ALS, rising in blood and cerebrospinal fluid months to years before phenoconversion in SOD1 and C9orf72 mutation carriers (Benatar et al., 2023; Goutman et al., 2022b; Gaetani et al., 2019; Delaby et al., 2020; Bridel et al., 2019; Verde et al., 2024; Witzel et al., 2024). However, NfL reflects structural axonal injury rather than transport dysfunction per se and lacks mechanistic specificity, being elevated across multiple neurological diseases (Gaetani et al., 2019; Delaby et al., 2020; Bridel et al., 2019; Verde et al., 2024). Given that NfL is released during axonal breakdown, it likely represents a downstream marker of neuronal injury rather than an indicator of early transport impairment. Moreover, NfL levels tend to plateau after symptom onset, suggesting that they track cumulative damage rather than the evolving dynamics of transport failure (Bridel et al., 2019; Verde et al., 2024; Witzel et al., 2024). There is therefore a need for biomarkers that more directly reflect axonal transport integrity.

Exosomal microRNAs regulating cytoskeletal dynamics, motor proteins, and trafficking pathways represent promising candidates. Multiple studies have identified dysregulated exosomal miRNAs in ALS—including miR-146a-5p, miR-23a-3p, miR-15a-5p, and miR-193a-5p—some of which regulate transport-related genes and show early alterations in disease (Kim et al., 2023; Rawat et al., 2025; Rizzuti et al., 2022; Saucier et al., 2019; Liu et al., 2023; Cheng et al., 2023). Although current evidence remains largely cross-sectional and requires longitudinal validation, these candidates are mechanistically aligned with transport biology.

A multimodal biomarker strategy integrating NfL (injury burden), exosomal cargo and miRNA profiles (mechanistic signaling), and advanced neuroimaging of corticospinal tracts may provide the temporal and mechanistic resolution necessary to detect presymptomatic transport dysfunction and monitor therapeutic response (Benatar et al., 2023; Bono et al., 2025; Roy et al., 2025).

## Therapeutic implications

Current ALS therapies primarily target downstream consequences of neurodegeneration. Riluzole modulates glutamatergic neurotransmission, and edaravone reduces oxidative stress, but neither directly addresses upstream disruptions in intracellular trafficking (Burk and Pasterkamp, 2019; Berth and Lloyd, 2023; Sever et al., 2022). This mechanistic distinction may partly contribute to their modest clinical impact.

By contrast, restoration of axonal transport improves transport metrics and extends survival in experimental models. Inhibition of p38 MAPK rescues retrograde transport defects and prolongs survival in SOD1 mice (Gibbs et al., 2018), while modulation of the GDNF-RET pathway normalizes axonal trafficking in diseased motor neurons (Rhymes et al., 2022). HDAC6 inhibition reverses transport deficits in FUS-ALS patient-derived motor neurons (Guo et al., 2017). These interventions act directly on transport machinery rather than solely mitigating downstream consequences.

Recent mechanistic studies demonstrate that p38α MAPK phosphorylates TDP-43 at pathogenic sites, promoting aggregation, and that its inhibition reduces toxicity in patient-derived motor neurons (Aikio et al., 2025). Brain-penetrant p38α inhibitors and emerging combinatorial strategies further support the translational relevance of targeting upstream transport pathways (Roy et al., 2019; Valipour et al., 2024; Jiang et al., 2022; Johnson et al., 2022; Lee et al., 2023; Bye et al., 2025).

We therefore propose that restoration of axonal transport may serve as a central component of rational multimodal ALS therapy, integrated with strategies targeting excitotoxicity, mitochondrial dysfunction, and neuroinflammation.

## Testable predictions

The hypothesis that axonal transport impairment is an upstream mechanism in ALS pathogenesis generates several testable predictions:

1. Presymptomatic detection of transport dysfunction: Axonal transport deficits should be measurable in at-risk individuals before clinical symptoms or structural axonal loss, using physiological assays such as *in vivo* imaging of retrograde transport or advanced neuroimaging techniques. This is supported by evidence that transport impairment is detectable in presymptomatic ALS models and mutation carriers (Bilsland et al., 2010; Lee et al., 2022; Feldman et al., 2022).
2. Temporal dissociation between transport failure and structural axonal loss: There should be cases where transport dysfunction precedes axonal degeneration, and conversely, axonal degeneration can occur without prior transport impairment. This prediction is validated by studies showing independent evolution of transport deficits and axon degeneration in ALS mouse models (Marinkovic et al., 2012).
3. Genotype-specific positioning of transport impairment in the pathogenic cascade: The timing and severity of transport dysfunction relative to other pathological events should vary by ALS genotype. For example, TDP-43 and SOD1 models show early transport deficits, while some FUS models do not, indicating genotype-modulated vulnerability (Bilsland et al., 2010; Guo et al., 2020; Marinkovic et al., 2012).
4. Correlation of selective vulnerability with dependence on long-range trafficking logistics: Neurons with greater reliance on long-range axonal transport, such as motor neurons, should exhibit earlier and more severe degeneration, while neurons with less dependence (e.g., sensory neurons) are relatively spared. This is observed in both animal and human studies (Baldwin et al., 2016; Ikenaka et al., 2012).
5. Therapeutic restoration of axonal transport modifying early disease trajectories: Interventions that restore axonal transport should delay or prevent downstream synaptic failure, bioenergetic stress, and motor neuron loss, and show benefit when applied presymptomatically or early in disease. Experimental therapies targeting transport machinery have demonstrated such effects in ALS models (Rhymes et al., 2022).
6. Performance of multimodal biomarker panels reflecting transport integrity compared to single injury markers: Biomarker panels incorporating transport-specific measures (e.g., exosomal miRNAs, imaging of axonal transport) should outperform single injury markers like neurofilament light chain (NfL) in detecting early disease, tracking progression, and predicting therapeutic response. NfL is sensitive but lacks mechanistic specificity, and multimodal approaches are recommended to improve diagnostic and prognostic precision (Feldman et al., 2022; Obara et al., 2025; Shefner et al., 2022).

## Limitations

Animal models of ALS may introduce selection bias, as commonly used systems emphasize specific genetic mutations and may not fully capture the heterogeneity of sporadic disease. Moreover, temporal relationships observed in transgenic models may not precisely reflect human disease progression.

Importantly, axonal transport impairment is not uniformly primary across all molecular subtypes. For example, certain FUS models do not demonstrate early retrograde transport deficits despite motor neuron loss (Sleigh et al., 2020), indicating genotype-specific variability in temporal ordering. Thus, transport dysfunction may represent a frequent but not universal upstream vulnerability.

In addition, current human evidence for early transport failure relies largely on indirect measures, including iPSC-derived motor neurons and neuroimaging correlates, rather than direct *in vivo* assessment of axonal trafficking dynamics.

Despite these limitations, convergent findings across species, mutations, and experimental systems, including rescue of FUS-ALS motor neurons by HDAC6 inhibition (Guo et al., 2017), disruption of RNA granule transport by FUS mutations (Goutman et al., 2022a), transport defects in TDP-43 and FUS Drosophila models (Baldwin et al., 2016), and suppression of mitochondrial trafficking pathways in human TARDBP- and FUS-ALS motor neurons (Schweingruber et al., 2025), support the plausibility of transport impairment as a central vulnerability in ALS. Nevertheless, definitive validation will require longitudinal human studies capable of directly quantifying transport dysfunction prior to neurodegeneration.

## Conclusion

Across genetic, cellular, and human systems, axonal transport impairment emerges as a recurrent early systems-level disturbance observed across many ALS models, though its position in the pathogenic cascade varies by genotype. By disrupting the delivery of mitochondria, mRNAs, and synaptic components, and by impairing retrograde clearance of damaged cargo, transport dysfunction may initiate distal synaptic collapse, bioenergetic stress, altered excitability, protein aggregation, neuroinflammation, and ultimately neuronal death.

This transport-centered framework provides a mechanistic explanation for selective motor neuron vulnerability, the dying-back pattern of degeneration, and the limited efficacy of current downstream-targeted therapies. Selective vulnerability in ALS is best understood as the interaction of axon length, neuronal identity, excitability state, and metabolic-transport coupling, rather than axon length alone.

Targeting axonal transport may therefore represent a rational strategy for early disease modification and biomarker-guided intervention. Importantly, this framework represents a testable mechanistic hypothesis rather than a definitive causal model.
